# Screening for Sleep Apnoea in Mild Cognitive Impairment: The Utility of the Multivariable Apnoea Prediction Index

**DOI:** 10.1155/2014/945287

**Published:** 2014-01-16

**Authors:** Georgina Wilson, Zoe Terpening, Keith Wong, Ron Grunstein, Louisa Norrie, Simon J. G. Lewis, Sharon L. Naismith

**Affiliations:** ^1^Healthy Brain Ageing Clinic, Brain & Mind Research Institute, University of Sydney, 94 Mallett Street, Camperdown, NSW 2050, Australia; ^2^Woolcock Institute of Medical Research, University of Sydney, Glebe, NSW 2050, Australia

## Abstract

*Purpose*. Mild cognitive impairment (MCI) is considered an “at risk” state for dementia and efforts are needed to target modifiable risk factors, of which Obstructive sleep apnoea (OSA) is one. This study aims to evaluate the predictive utility of the multivariate apnoea prediction index (MAPI), a patient self-report survey, to assess OSA in MCI. *Methods*. Thirty-seven participants with MCI and 37 age-matched controls completed the MAPI and underwent polysomnography (PSG). Correlations were used to compare the MAPI and PSG measures including oxygen desaturation index and apnoea-hypopnoea index (AHI). Receiver-operating characteristics (ROC) curve analyses were performed using various cut-off scores for apnoea severity. *Results*. In controls, there was a significant moderate correlation between higher MAPI scores and more severe apnoea (AHI: *r* = 0.47, *P* = 0.017). However, this relationship was not significant in the MCI sample. ROC curve analysis indicated much lower area under the curve (AUC) in the MCI sample compared to the controls across all AHI severity cut-off scores. *Conclusions*. In older people, the MAPI moderately correlates with AHI severity but only in those who are cognitively intact. Development of further screening tools is required in order to accurately screen for OSA in MCI.

## 1. Introduction

Mild cognitive impairment (MCI) is a syndrome defining a transitional stage between normal ageing and dementia. Clinically, it is defined as cognitive decline greater than expected for an individual's age and education, but with preservation of daily functioning [1]. Since there is a conversion rate to dementia of around 50% in five years, MCI is often considered an “at risk” state. Importantly, in this critical period, there is opportunity to implement secondary prevention strategies targeting modifiable risk factors. Research to date has identified that a range of cardiovascular, psychological, and lifestyle factors are associated with an increased conversion to dementia. However, there has been a paucity of research addressing sleep. This is despite the fact that sleep disturbance is a common symptom of dementia [2], associated with decreased cognitive and daily functioning, reduced quality of life, and increased carer burden [3].

Sleep disturbance in older people is multifaceted and includes age-related changes to sleep macro- and microarchitecture, medical comorbidity, mood disturbance, and alterations in circadian rhythm [3, 4]. In addition, the prevalence of nocturnal respiratory disturbance increases with age, and, in particular, obstructive sleep apnoea (OSA) affects 70% of men and 56% of women over the age of 65 years [5], a figure which is markedly increased from that observed in the general adult population, where the prevalence is only around 2–7% [6]. OSA is characterised by repetitive partial or complete collapse of the pharyngeal airway during sleep and can affect brain functioning in many ways including intermittent hypoxemia, sleep fragmentation, and consequent hypersomnolence. The prevalence of OSA in older adults with a neurodegenerative disorder is less well understood. Studies have shown that up to 40% of institutionalised Alzheimer's disease (AD) patients suffer from OSA [7] and that untreated OSA can exacerbate the primary cognitive and functional deficits associated with this disorder. In MCI, there are few detailed studies, but some data suggests that OSA may be linked to impaired language function [8].

Although research investigating links between OSA and dementia are in their infancy, there is mounting evidence linking OSA to cognitive decline in younger cohorts with poorer performance in the domains of processing speed, attention, learning/memory, and executive functioning [9, 10]. Moreover, longitudinal studies have further confirmed that OSA is a predictor of cognitive decline [11]. Pathophysiologically, there appear to be at least two primary contributors to cognitive decline, namely, hypoxic brain changes due to oxygen desaturation (associated with repetitive apnoeas or hypopnoeas) and/or sleep fragmentation due to frequent arousals or awakenings. It has been postulated that hypoxemia and sleep fragmentation contribute differentially to neuropsychological dysfunction in OSA, with the former being specifically linked to executive deficits and the latter being linked to changes in processing speed [9, 12]. Disruption to sleep microarchitecture from sleep fragmentation may also impede sleep-related memory consolidation [13].

Treatment for OSA, such as continuous airway pressure (CPAP), has been shown to decrease sleep disturbance in patients with AD and OSA [14] and, although the cognitive benefits of CPAP are not yet clear, some studies have shown positive improvement in neuropsychological functioning [7, 15]. Overall, there is evidence suggesting that, even in established dementia, there may be merit in addressing this problem and this may be even greater if intervention targeted critical “at risk” periods such as MCI. In order to effectively detect OSA at the population level, it is necessary to have effective screening tools. Currently, overnight polysomnography (PSG) represents the gold standard for evaluating respiratory disturbance providing detailed data on airflow, oxygen saturation, and sleep cycle. It is, therefore, an accurate diagnostic tool for OSA. The apnoea-hypopnoea index (AHI: number of apnoeas/hypopnoeas per hour of sleep) is commonly used as an indicator of OSA severity. However, there are many barriers to the assessment of patients in overnight sleep laboratory facilities. The process is costly and requires qualified staff to run and interpret complex data, which is often time consuming and associated with long waiting lists [6]. Older people may also be reluctant to leave their homes and often an adaptation night is required in order to capture the usual sleep pattern [16]. Therefore, there has been a drive to develop validated screening instruments for OSA that are easily administered and cost-effective.

One of the common screening tools that has been developed for use in OSA generally is the multivariable apnoea prediction (MAP) survey which predicts apnoea risk as a score between 0 (low risk) and 1 (high risk) termed the multivariate apnoea prediction index (MAPI) [17]. The predictive ability of the MAPI was determined by receiver operating characteristics (ROC) curve analysis offering good sensitivity and specificity for the detection of respiratory disturbance [17] in younger cohorts, where the optimum MAPI value was determined to be 0.50. The MAPI has been validated for use in sleep and nonsleep disorder clinical samples and primary care settings, in order to discriminate between people likely and unlikely to have OSA [18–20]. Also, in one elderly sample with excessive daytime sleepiness, the MAPI demonstrated a predictive utility that was comparable to that obtained in sleep disorder clinic samples. In addition, it was also identified that, compared to BMI alone (which by itself is a very strong predictor of OSA in adult samples), the symptom questions on the survey were necessary to obtain adequate predictive value in the older population with excessive daytime sleepiness [19].

To our knowledge, no study has previously examined the utility of the MAP questionnaire in those patients with MCI who are “at risk” of dementia. We proposed that, if the MAPI was found to be a reliable method for screening OSA in this cohort, it may be an effective method of screening older people for OSA and may negate the need for formal overnight PSG studies. Therefore, the main objective of this study was to determine the predictive utility of the MAPI for determining AHI in older people and specifically in patients with MCI.

## 2. Method

### 2.1. Patients

Thirty-seven older adults over 50 years of age meeting Petersen's criteria for MCI [21] (mean age = 65.5 years, SD = 9.0) were recruited from the Healthy Brain Ageing Clinic at the Brain and Mind Research Institute, University of Sydney, Australia. In accordance with these criteria, patients were classified as having MCI if they performed at 1.5 standard deviations below their predictive level of intelligence on at least one neuropsychological test. Thirty-seven age-matched controls were recruited from the community via local community advertisement (mean age = 63.5 years, SD = 8.7). Exclusion criteria included history of stroke, head injury with a loss of consciousness for more than 30 minutes; neurological disease (e.g., Parkinson's disease, epilepsy); psychotic illness; medical conditions known to affect cognition (e.g., cancer); mini-mental state examination (MMSE) score <24; diagnosis of dementia; illicit substance use; shift workers, transmeridian travel within 60 days before overnight laboratory assessment; and use of medication known to affect sleep and/or melatonin secretion including beta-blockers, lithium or benzodiazepines or sedative hypnotics. Prior to overnight PSG assessment, patients were also required to abstain from alcohol and caffeinated drinks for 48 and 24 hours respectively.

### 2.2. Clinical Assessment

All controls and patients received comprehensive clinical assessments by an old age psychiatrist who interviewed patients regarding medical history including current medications, heart disease, hypertension, and diabetes. Physical measurements including height, weight, and BMI were also taken. The cumulative illness rating scale (CIRS) [22] total score (the sum of all the scores of all categories) was used to evaluate overall medical burden. Global cognitive functioning was measured by the mini-mental state examination (MMSE) [23]. As described elsewhere [24], all participants were further assessed by a clinical neuropsychologist, who confirmed MCI diagnosis and that the patient did not meet criteria for dementia. Individuals were also diagnosed with multi- or single domain MCI (depending on if there were deficits in more than one cognitive domain) and amnestic (only memory impairment) or nonamnestic MCI [25]. This study was approved by the University of Sydney Institutional Ethics committee and all participants gave written informed consent.

### 2.3. Measures

#### 2.3.1. Polysomnography

All participants underwent standard nocturnal PSG which included electroencephalography (EEG), electrooculography (EOG), electromyography (EMG), and pulse oximetry. A subset of participants (MCI = 24; controls = 25) also had nasal airflow (using a nasal pressure transducer or thermistor) and respiratory effort (using thoracic and abdominal bands) measured which enabled AHI to be calculated. Sleep architecture stages were scored by an experienced sleep technician using standardised criteria [26] modified for older subjects [27]. The main outcome variables used in this study included AHI (obtained for a subset of participants), oxygen desaturation index (ODI), and average oxygen desaturation (obtained for all participants). Apnoea was defined as complete cessation of airflow on the nasal pressure transducer or thermistor for at least 10 seconds. Hypopnoea was defined as a reduction of nasal airflow >50% with either a 3% desaturation or an arousal [28]. AHI was calculated as the total number of apnoeas and hypopnoeas divided by the total sleep time, giving the number of apnoeas/hypopnoeas per hour of sleep. ODI was calculated as the number of oxygen desaturations >3% per hour of sleep.

#### 2.3.2. Self-Report

*(a) Multivariable Apnoea Prediction Survey [17].* All participants completed the MAP survey, which predicts apnoea risk using demographic data and self-reported apnoea symptoms. Three frequency questions as well as gender, age, height, and weight (to calculate BMI) are used to produce a MAPI between 0 (low risk) and 1 (high risk). The three questions determine the frequency that the patient has experienced loud snoring, snoring or gasping, cessation of breathing, or struggle for breath in the last month. Together, the questions produce a score referred to as Index 1. 

*(b) The Pittsburgh Sleep Quality Index (PSQI) [29]*. This was used to determine an overall subjective measure of sleep quality over the previous month. The questionnaire consists of 17 items with most questions rated on a 4-point Likert scale. The PSQI provides a global score based on the components of quality, latency, duration, efficiency, disturbance, use of sleeping medication, and daytime dysfunction.

### 2.4. Statistical Analyses

Statistical analysis was conducted using the statistical package for social sciences (SPSS) version 20. Independent samples *t*-tests were used for normally distributed variables to compare the descriptive statistics for controls versus MCI patients. To investigate the association between BMI and OSA in our whole sample, independent *t*-tests were used with AHI ≥ 5. Chi-square tests were used for categorical variables, except where Fisher's exact test was used to analyse medical conditions including heart disease, hypertension, and diabetes. Pearson correlations were employed to examine the associations between normally distributed variables and all other correlations used Spearman rank. All analyses were two-tailed with an alpha level of 0.05.

To determine the predictive utility of the MAPI in our sample of older adults, receiver-operating characteristics (ROC) curve analysis was performed using the program *MedCalc* version 12.2.1.0. ROC curves were generated using cutoff scores of AHI ≥ 5, AHI ≥ 15 and AHI ≥ 30. These particular cut-offs were chosen on the basis of the values typically used to classify severity of OSA in older adults [28]. The sensitivity (true positive rate) is plotted in function of 1-specificity (false positive rate). The Youden index, defined as “sensitivity + specificity − 1,” was used to determine the optimal MAPI cut-off points in each analysis, where equal weight was given to the sensitivity and specificity of the test.

## 3. Results

Descriptive data for the sample including clinical and sleep variables is presented in Table 1. Of the 37 MCI patients, 27 were of the nonamnestic type and 10 had amnestic MCI. Twenty-eight MCI patients had multiple cognitive domains affected and nine had only a single domain. No significant differences in terms of age, BMI, height, or weight were identified between the controls and those with MCI. As expected, the MMSE scores were significantly lower in the MCI sample (*P* < 0.01). Those with MCI had a significantly higher medical burden as evidenced by the CIRS total score (*P* = 0.012). No significant differences were found between the MCI and control groups in terms of diagnosis of hypertension (*P* = 0.33), diabetes (*P* = 0.26), or heart disease (*P* = 0.67). For the whole sample, no significant differences were found regarding BMI and OSA (AHI ≥ 5: *t*-value (df) = −0.483 (47), *P* = 0.63).

Of the control sample with AHI data, 68.0% had an AHI ≥ 5; 28.0% AHI ≥ 15, and 8.0% AHI ≥ 30. Within the MCI sample with AHI data, 70.8% had an AHI ≥ 5; 41.7% AHI ≥ 15; and 16.7% AHI ≥ 30. Table 1 shows that the two groups did not differ significantly in terms of self-reported sleep quality (PSQI) or PSG-derived apnoea measures. There was a trend for a greater proportion of controls (54.2%) to have central sleep apnoea (CSA) compared to those with MCI (26.1%) (*χ* ^*2*^ (1) = 3.45, *P* = 0.05).

As expected, AHI correlated significantly with ODI and average oxygen desaturations in both the MCI (*r* = 0.59, *P* = 0.002 and *r* = 0.78, *P* < 0.001, resp.) and control (*r* = 0.78, *P* < 0.001 and *r* = 0.72, *P* < 0.001, resp.) group (not shown in the table).

*Correlation of MAPI with Apnoea Measures.* Table 2 shows that, in the control group, a modest but significant correlation was observed between higher MAPI scores and more severe apnoea (AHI) (*P* = 0.017) as well as more frequent oxygen desaturations (ODI) (*P* = 0.010). However, the correlation in the MCI sample was nonsignificant. There was also a significant difference between the correlation coefficients for MAPI and AHI between the MCI and control sample (Fishers *r* to *z* transformation, *z* = 2.15, *P* = 0.032), although this was not found between the MAPI and ODI correlation coefficients.

Interestingly, MAPI and age were inversely correlated in the MCI sample only (Table 2). As this finding is unusual, the relationship between age and BMI (a major predictor in the MAPI) was investigated in the MCI sample. These variables were also found to be inversely correlated, but this relation was not statistically significant (*r* = −0.11, *P* = 0.525). 

*ROC Curve Analysis.* Table 3 displays the ROC curve data for the whole sample, MCI, and control participants and Table 4 shows the area under the curve (AUC) data. Table 3 indicates the specificity and sensitivity for the optimal cut-off MAPI scores to various AHI scores as determined by ROC curve analysis. When detecting any degree of OSA (i.e., AHI ≥ 5) in the whole sample, the optimum cut-off score for older adults on the MAPI was 0.29 with a sensitivity of 67.65% and specificity of 60.00%. Rather surprisingly, when detecting severe OSA (i.e., AHI ≥ 30), the optimal cut-off score was paradoxically lower, at 0.15, which afforded improved sensitivity (100.00%) but at a trade-off for specificity (18.60%). However, in our sample, only few participants were diagnosed with severe OSA (MCI: 4; controls: 2). To detect mild OSA (AHI ≥ 15), the MAPI cut-off score (0.21) is also lower than AHI ≥ 5 with an improved sensitivity of 88.24% but lower specificity of 31.25%.

In control participants, the AUC was greater at every cut-off score of AHI as compared to the corresponding findings in the MCI sample (Table 3). The optimum cut-off score for suspicion of OSA (AHI ≥ 5) in the MCI sample was much higher (MAPI of 0.65) compared to controls (MAPI of 0.27) with decreased specificity. In the MCI group, when detecting severe OSA (AHI ≥ 30), the cut-off score was lower (0.27) than was found in controls (0.33). For detecting mild OSA (AHI ≥ 15), the MAPI cut-off score in the MCI sample (0.56) was higher compared to the control (0.22) and whole sample (0.22).

The AUCs for MAPI, Index 1, and BMI were also compared simultaneously between MCI and controls (data not shown). In the MCI sample, there was a reduced ability of Index 1 to discriminate between an AHI ≤ 5 and AHI ≥ 5 (AUC = 0.353). There was also a significant positive correlation found between Index 1 and overall MAPI score in the MCI group (*r* = 0.353, *P* = 0.048), a relationship that was not observed in controls.

## 4. Discussion

This is the first study to investigate the utility of the MAP survey in patients with MCI and in comparison to healthy older controls. The results show that, in this sample of patients, the use of the MAPI to predict sleep apnoea offers significantly reduced utility as compared to studies in other age groups and clinical samples.

An analysis of this entire sample of older people found that, to detect severe sleep apnoea (AHI ≥ 30), the MAPI cut-off score was determined to be lower than all other severities of OSA, a finding which is in contrast to that expected. However, it is noted that only small numbers of participants had an AHI above this cutoff, thus preventing valid conclusions to be drawn regarding the utility of the tool for severe OSA.

Importantly, however, analysis of subsamples of MCI patients and controls showed that there was a significant difference in correlations between AHI and MAPI amongst controls versus MCI, suggesting that the relationship between MAPI and AHI differs according to the presence of cognitive impairment. Specifically, ROC curve analysis also showed that the MAPI is less valid in the MCI sample compared to healthy controls, with consistently lower AUC irrespective of the cutoff used in the MCI group. While it is not possible from this study alone to definitively ascertain why the MAPI is unsuitable for those with MCI, we suspect it is due to MCI patients having poor recall of sleep-wake disturbances and/or failing to report their symptoms due to their cognitive impairments. This was supported by our AUC data, which found a reduced ability of Index 1 of the MAPI to detect the presence of OSA (as measured by AHI) in the MCI group, which was not evident in controls. Another possible explanation could be that the factors that load heavily on the MAPI may not have great utility within an MCI or an older adult population. Although no significant difference emerged in common conditions associated with OSA (hypertension, heart disease, or diabetes) between the MCI and control groups, the CIRS indicated that the MCI group did have higher medical burden. Also, BMI is a factor which loads heavily on the MAPI; however, within the whole sample, there was no significant difference found between BMI and people with or without OSA (defined as AHI ≥ 5 or AHI ≥ 10). Therefore, rather than factors traditionally used to derive OSA risk such as BMI, other factors may be pertinent to the development of sleep apnoea in this age group, such as upper airway collapsibility from changes in structures surrounding the pharynx or age-related differences in respiratory control. It was also interesting to find that MAPI and age were inversely correlated in the MCI sample, as were age and BMI. Increasing BMI and age are important risk factors for OSA in the normal population; therefore, this unusual finding in our MCI sample could provide some explanation for the nonsignificant results in this subset of participants. PSG-confirmed AHI would not consider age or BMI whereas the MAPI is heavily weighted on these risk factors. Therefore, AHI may be more reliable in this group.

The MAP survey has been specifically derived to detect OSA raising the possibility that our MCI sample may actually be suffering from other sleep disorders such as CSA. Whilst having an elevated AHI on PSG, this disturbance would not be registered by the MAP survey. The pathophysiology underlying CSA is related to a failure in signalling from the brain to the muscles of ventilation resulting in cessation of breathing [30]. Unlike OSA, CSA does not trigger increased respiratory effort to breathe, and therefore loud snoring (a key component of the MAPI) is not a common symptom. However, CSA is not more prevalent in our MCI sample suggesting this is not a factor affecting the results found.

Evidently, our findings suggest that PSG remains the most reliable method for evaluating OSA in patients with MCI. Other self-report questionnaires such as the Epworth sleepiness scale have also been shown to only correlate poorly with AHI [31] suggesting that symptomatology is not always concordant with apnoea severity. However, future studies could be focused on alternative methods of PSG, such as home monitoring devices, which might prove both efficacious and well tolerated in MCI cohorts [32, 33]. In conclusion, although the MAP survey is an inexpensive and easy-to-administer screening instrument, its poor specificity reduces its clinical utility for patients with MCI.

## Figures and Tables

**Table 1:** A comparison of clinical, self-report, and polysomnographic data between MCI and control subjects.

	Control Mean ± SD	MCI Mean ± SD	*t* value (df)/*z*	*P* value
Clinical				
Age (years)	63.5 ± 8.7	65.5 ± 9.0	−0.98 (71)	0.333
Body mass index	27.1 ± 4.0	27.6 ± 5.5	−0.43 (72)	0.669
Weight (kg)	75.9 ± 14	78.0 ± 16	−0.60 (72)	0.552
Height (cm)	167 ± 10	168 ± 11	−0.43 (72)	0.668
CIRS, total score	3.0 ± 2.3	5.9 ± 6.4	−2.57 (67)	0.012*
MMSE score	29.2 ± 1.1	28.1 ± 1.5	3.75 (72)	<0.001**
Self-report				
MAPI	0.3 ± 0.2	0.4 ± 0.2	−1.30 (72)	0.199
PSQI, global score	5.4 ± 4.0	7.0 ± 3.4	−1.83 (69)	0.072
PSQI, sleep efficiency (%)	77.6 ± 10	75.5 ± 14	0.63 (68)	0.533
Overnight polysomnography				
Apnoea-hypopnoea index	11.9 ± 10	16.4 ± 16	−1.20 (47)	0.236
Total sleep time (minutes)	377 ± 73	385 ± 100	−0.41 (72)	0.682
Sleep efficiency (%)	77.6 ± 10	75.6 ± 14	0.73 (72)	0.468
WASO (mins)	96.9 ± 53	97.9 ± 56	−0.08 (72)	0.938
Lowest oxygen desaturation (%)	87.6 ± 5.0	87.9 ± 8.4	−0.19 (72)	0.854
Average oxygen desaturation (%)	4.1 ± 1.4	6.5 ± 13	−1.10 (72)	0.273
Oxygen desaturation index	23.9 ± 21.6	39.3 ± 65.4	−1.21	0.228
Non-REM sleep AHI	9.5 ± 9.9	14.2 ± 15	−1.31 (47)	0.198
REM sleep AHI	18.6 ± 15	19.8 ± 16	−0.11 (47)	0.916

*P < 0.05, **P < 0.01.

MCI: mild cognitive impairment; CIRS: cumulative illness rating scale; MMSE: mini-mental state examination; MAPI: multivariable aponea prediction index; PSQI: pittsburgh sleep quality index; AHI: apnoea-hypopnoea index; WASO: wake after sleep onset; REM: rapid eye movement.

**Table 2:** Correlation coefficients between the MAPI and clinical and polysomnographic data for controls (*n* = 37) and patients with mild cognitive impairment (MCI) (*n* = 37).

	MAPI Controls	MAPI MCI
Age	0.458**	−0.165
Body mass index	0.491**	0.499**
MMSE score	−0.086	−0.084
CIRS, total score	0.325	0.287
Apnoea-hypopnoea index (nonparametric test)	0.474* (*n* = 25)	−0.141 (*n* = 24)
Average oxygen desaturation (non-parametric test)	0.279	−0.144
Oxygen desaturation index (non-parametric test)	0.418**	−0.077
Lowest oxygen desaturations	−0.308	−0.180

*P < 0.05, **P < 0.01.

MAPI: multivariable apnoea prediction index; CIRS: cumulative illness rating scale; MMSE: mini-mental state examination.

**Table 3:** ROC curve analysis demonstrating optimal MAPI scores according to various apnoea-hypopnoea index (AHI) scores for the whole sample, MCI, and control sample.

	Whole sample (*n* = 49)			MCI sample (*n* = 24)			Control sample (*n* = 25)		
	AHI ≥ 5	AHI ≥ 15	AHI ≥ 30	AHI ≥ 5	AHI ≥ 15	AHI ≥ 30	AHI ≥ 5	AHI ≥ 15	AHI ≥ 30
AUC	0.567	0.580	0.502	0.576	0.504	0.650	0.699	0.671	0.761
Sensitivity	67.65*	88.24*	100.00*	88.24*	20.00*	75.00*	82.35*	100.00*	100.00*
Specificity	60.00*	31.25*	18.60*	42.86*	64.29*	70.00*	75.00*	38.89*	52.17*
MAPI cutoff	(0.29)	(0.21)	(0.15)	(0.65)	(0.56)	(0.27)	(0.27)	(0.22)	(0.33)

ROC: receiver operating characteristic; MAPI: multivariable aponea prediction index; AHI: apnoea-hypopnoea index; AUC: area under the curve. *Criterion corresponding with the highest Youden index.

**Table 4:** ROC curve analysis demonstrating the area under the curve (AUC) values according to various apnoea-hypopnoea index (AHI) scores for the whole sample (*n* = 48), control (*n* = 25), and MCI (*n* = 24) samples.

AHI	Sample	AUC (95% CI)
AHI ≥ 5	Whole	0.567 (0.323, 0.837)
	Control	0.699 (0.349, 0.968)
	MCI	0.576 (0.099, 0.816)

AHI ≥ 15	Whole	0.580 (0.161, 0.500)
	Control	0.671 (0.173, 0.643)
	MCI	0.504 (0.351, 0.872)

AHI ≥ 30	Whole	0.502 (0.084, 0.334)
	Control	0.761 (0.306, 0.732)
	MCI	0.650 (0.457, 0.881)

ROC: receiver operating characteristic; AHI: apnoea-hypopnoea index; AUC: area under the curve; MCI: mild cognitive impairment.
